# Perspectives and history in genodermatoses

**DOI:** 10.1111/1346-8138.17657

**Published:** 2025-02-20

**Authors:** Akiharu Kubo

**Affiliations:** ^1^ Division of Dermatology, Department of Internal Related Kobe University Graduate School of Medicine Kobe Japan

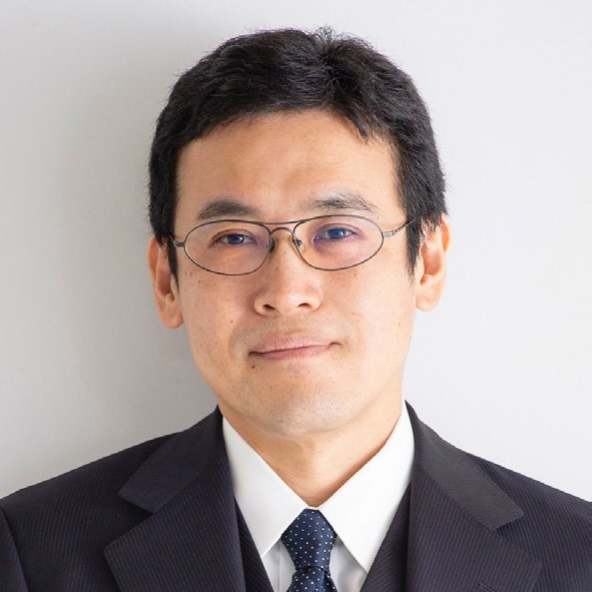

Advances in next‐generation sequencing have facilitated the identification of disease‐associated genes for a significant proportion of genetic skin diseases, thereby integrating genetic diagnosis into standard medical care. In Japan, the list of genetic diseases for which genetic diagnosis is available under national health insurance is expanding every year. Since misinterpretation of results of genetic analyses can lead to incorrect genetic counseling, the need for physicians to have a thorough knowledge of genetics is growing massively.

A common challenge in genetic diagnosis is determining whether multiple heterozygous variations detected in the proband are restricted to a single allele or are present in different alleles. This challenge is called “phasing” and typically arises when genomic DNA from the proband's parents is not available. Natsuga presents a comprehensive review of phasing, a critical method for addressing this challenge.

It is becoming clear that a variety of genetic factors underlie common diseases such as atopic dermatitis and psoriasis. Akiyama provides an update on the genetic basis of pustular psoriasis, which is thought to have a particularly strong genetic component associated with inflammatory responses.

There are still some rare diseases for which the genetic cause has not been identified, even with the latest various genetic analysis methods, including next‐generation sequencing. To identify a novel disease and a novel genetic, epigenetic or yet unknown genomic cause, it is necessary to accumulate as many cases of the disease as possible. For this purpose, careful description of skin lesions by descriptive dermatology and systematic search for analogous cases is important. Kubo described the history of the discovery of Nagashima‐type palmoplantar keratosis from the identification of the disease to the identification of the disease‐causing variants in SERPINB7. This record will be useful for future attempts to identify a new independent disease and its pathogenetic factors.

